# Patient safety culture in care homes for older people: a scoping review

**DOI:** 10.1186/s12913-017-2713-2

**Published:** 2017-11-21

**Authors:** Emily Gartshore, Justin Waring, Stephen Timmons

**Affiliations:** Centre for Health Innovation Leadership and Learning, Nottingham Business School, Jubilee Campus, Nottingham, NG8 1BB UK

**Keywords:** Care home, Residential home, Nursing home, Safety culture, Organisational culture, Scoping review, Scoping study

## Abstract

**Background:**

In recent years, there has been an increasing focus on the role of safety culture in preventing incidents such as medication errors and falls. However, research and developments in safety culture has predominantly taken place in hospital settings, with relatively less attention given to establishing a safety culture in care homes. Despite safety culture being accepted as an important quality indicator across all health and social care settings, the understanding of culture within social care settings remains far less developed than within hospitals. It is therefore important that the existing evidence base is gathered and reviewed in order to understand safety culture in care homes.

**Methods:**

A scoping review was undertaken to describe the availability of evidence related to care homes’ patient safety culture, what these studies focused on, and identify any knowledge gaps within the existing literature. Included papers were each reviewed by two authors for eligibility and to draw out information relevant to the scoping review.

**Results:**

Twenty-four empirical papers and one literature review were included within the scoping review. The collective evidence demonstrated that safety culture research is largely based in the USA, within Nursing Homes rather than Residential Home settings. Moreover, the scoping review revealed that empirical evidence has predominantly used quantitative measures, and therefore the deeper levels of culture have not been captured in the evidence base.

**Conclusions:**

Safety culture in care homes is a topic that has not been extensively researched. The review highlights a number of key gaps in the evidence base, which future research into safety culture in care home should attempt to address.

## Background

Since the term ‘safety culture’ first emerged in 1988 following the nuclear energy Chernobyl disaster, it has become a commonly used term and has received varying definitions. Safety culture is defined by the Health and Safety Executive (1993) to be “the product of individual and group values, attitudes, perceptions, competencies, and patterns of behaviour that determine the commitment to, and the style and proficiency of, an organization’s health and safety management” [1]. Following the Institute of Medicine Report “To Err is Human”, patient safety emerged as an international health policy priority in the early 2000s [2, 3]. Since then, major failures in care [4–9] and landmark reports [10–12] have highlighted the influence of organisational factors and the need for a patient safety culture. However, the concept of ‘safety culture’ remains widely debated across the literature, with varying definitions across disciplines and theoretical assumptions [13].

Organisational ‘culture’ is often related to the organisational ‘climate’ and both terms are often described as necessary components for quality and safety within healthcare [13, 14]. These terms are often referred to in the literature without clear distinction. However, ‘culture’ studies often attempt to look at deeper levels than ‘climate’ studies, which usually look at surface perceptions and structures [15–17]. As culture has varying levels, which include surface artefacts that might be attributable to ‘climate’ [18–20], but also deeper levels of values and meaning that influence how social groups are organised and people interact, ‘safety culture’ will be used to encompass both throughout this paper.

Specifically, Schein (1988, 2004, 2010) identifies three levels for analyzing organisational culture, with changing degrees of visibility to the observer [18–20]. Level 1 is the superficial “Artifacts”, which are visible organisational structures and processes. The second level of analysis is “Espoused Beliefs and Values”, which are the conscious values, norms and rules, which individuals use to justify behavior and decisions. Through looking at organisational culture at these superficial levels, some understanding can be achieved, but this can arguably be misleading if the deepest level of culture is not also understood. The third level, “Basic Underlying Assumptions”, represents the deepest level for analyzing culture, and is seen to be where the essence of culture lies. This level of culture is often taken for granted and represent the deepest layer of culture. Schein (2004, p.36) states that “the essence of culture lies in the pattern of basic underlying assumptions, and once one understands those, one can easily understand the other more surface levels”.

There is an abundance of available evidence supporting the assessment and measurement of safety culture within mainstream healthcare settings [21–27], which includes frequent use of this as a quality and regulatory measure [28]. The most adopted assessment tool across hospital settings is the Agency for Healthcare Research and Quality- Hospital Survey on Patient Safety Culture (HSOPSC) [29], which aims to evaluate and improve safety culture. Much of the research applying such tools and frameworks has taken place within mainstream care settings, especially acute hospitals, historically led by the medical profession [3, 30]. However, safety culture is also important within non-mainstream care settings such as care homes, which have received far less attention in terms of research and safety development [36, 37]. Given that care homes provide care to over 450,000 people in the UK- more than 3 times the number of available NHS beds [31] – and that this care is typically provided to vulnerable older people, with functional dependence and cognitive impairment [32–34], it is vital that the safety of care within these settings is better understood. With growing evidence of variation in the quality and safety of care home care [38] there is recognition of the need for a positive safety culture within these settings for improving both quality and outcome [39].

An adapted version of the HSOPSC has been developed for care homes [40], although there does not appear to be evidence of how widely this has been adopted or how it is used by the care homes sector. Safety culture has also been used as a regulatory indicator within adult social care [41]. These approaches, which originated in acute hospital settings, have been directly transferred to the care home sector [39, 40]. This is particularly important, as care home organisations are in many ways different to acute care settings, and these differences can have significant implications for safety culture. A key difference is that the majority of care homes in England are residential (73%) [35]. Unlike Nursing Homes, Residential Homes provide long-term care to residents without any nursing provision, instead relying on a non-regulated workforce to deliver care [42], and community healthcare professionals to meet the nursing needs for the residents. This requires the complex working by multiple agencies, organisations and external stakeholders to ensure the safety of these older people [32–34]. Finally, care homes are unique in so far as the care setting is also the person’s home.

### Research focus

Despite safety culture being accepted as an important quality indicator across all health and social care settings, the understanding of culture within social care settings is far less well developed than within hospitals [13, 14, 36, 37]. It is therefore important that the existing evidence base is gathered and reviewed in order to understand this topic. To this end, a scoping review was conducted with the goals of describing the availability of evidence related to care homes’ patient safety culture, what these studies focused on, and identifying any knowledge gaps within the existing literature. All identified studies were included within the review and no quality constraints were applied [43, 44]. To the authors’ knowledge, few studies have shared the goal of this review. This study is therefore contributing to the growing evidence base on this topic. In scoping reviews, it is important that the overall purpose is identified and linked to the research questions [43, 44]. The research question this study aimed to answer was: What does the evidence base demonstrate in terms of methodology, theoretical underpinnings and specific focus of safety culture research in care homes?

## Methods

### Scoping review

A scoping review was used to answer the research question. Scoping reviews apply systematic principles to reviewing the literature for the purposes of: 1. Examining the extent, range and scope of research activity; 2. Addressing a broad review question; 3. Including all available research, irrespective of study design; 4. Providing a description of available evidence without assessing the quality of the reviewed studies [43–46]. This methodology has become increasingly popular and is used to identify gaps in the evidence, inform research agendas, policy and practice [47]. As such, a scoping review was an appropriate methodology to answer the research question, and has been used in a number of previous topics around healthcare safety [48–50].

### Search strategy and article selection

The database search was run on the 20th January 2017 by one of the authors (EG). Article selection and review took approximately one month and was completed on the 16th February 2017 by all authors. The keywords for this search were identified through engaging external expert advisors from the care home industry. Final terms were determined after an initial broad search using MEDLINE, which was used to identify MESH headings and alternative terms used in relevant papers. The keywords and MESH headings (patient safety, safety culture, safety climate, sense-making, long-term care, nursing home, care home, residential home and alternative spellings, plurals and related terms) were then used to search each database. The databases, MEDLINE, CINAHL, ASSIA, PsycINFO, EMBASE, and ProQuest Dissertation and Theses were searched to identify all relevant published and unpublished studies [33]. A narrowly defined search was used to maximise the relevance of papers and reduce the number of irrelevant studies that did not meet the inclusion criteria.

**Fig. 1:**
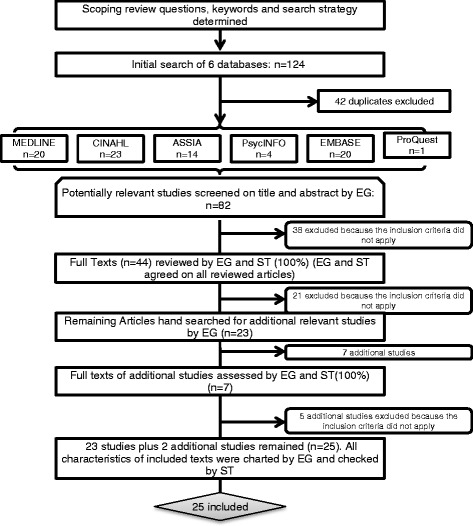
Selection process and search flow

Studies were only included if they were written in English. To fit the overall purpose of this scoping review, the included studies had to be based in a care home setting that provided residential or nursing care to older adults and needed to explore the phenomena of safety culture. Due to the scoping nature of this review, included studies could have any research methodology; grey literature and texts that were not peer reviewed were also included [43]. The inclusion criteria and search strategy are shown in Table 1. The selection process and search flow is demonstrated in Fig. 1.

**Table 1:** Inclusion criteria and search strategy

Inclusion criteria	- written in English - reports clinical research (is not commentary) - focus is on care home settings that provides 24-h long-term residential or nursing care to older adults - concerns safety culture
Search strategy in MEDLINE (Ovid)	Patient safety (MESH) AND Organizational culture (MESH) OR “safety culture*” OR “safety climate*” OR “sense making” OR sensemaking OR sense-making AND Nursing homes (MESH) OR Long-term care (MESH) OR Homes for the aged (MESH) OR “care home*” OR “residential home*”

### Data extraction

A spreadsheet was used to aid charting of the information to answer the research questions. Characteristics included publication details, setting, participants, research method, measurement tool, level of analysis, theoretical underpinnings and focus. This process was undertaken by one of the authors (EG). The extracted information was discussed during meetings with all authors in order to answer the research questions and describe the factors emerging from the literature. Any disagreements were discussed until a consensus was reached.

## Results

### Selection process and search flow

A total of 124 publications were initially identified from the databases, of these 42 duplicates were excluded. All abstracts were reviewed against the inclusion criteria by EG. The remaining 44 full texts were then reviewed by two authors (EG and ST) to reach a consensus on the included articles. During this process it was decided that a relevant literature review should be included within the study, as this text cited relevant papers and would then be screened for further full texts. Moreover, due to the limited availability of articles, four relevant papers that explored validation of safety culture surveys were also included to provide a broader overview of the available validation studies. 23 papers were included at this stage, and the full texts were screened to identify additional relevant papers (*n* = 7), that were again reviewed by two authors (EG and ST) to identify 2 further texts for inclusion. At the end of the selection process 24 empirical studies and 1 literature review [51] remained for further analysis (Fig. 1). Table 2 provides an overview of each included article; highlighting the setting of the study (primarily Nursing Homes and Residential Homes), participants (Frontline staff delivering direct care, or Administration/management staff), the research method, measurement tool, the level of analysis (according to Schein’s three levels of cultural analysis) and the focus of the article.

**Table 2:** Characteristics of included studies

First author, year, country	Setting	Participants	Research method	Measurement tool	Level of analysis	Focus
Arnetz, 2011, USA [52]	NH	Frontline & Administration	Quantitative Survey	NHSOPSC & Quality-work competence questionnaire	1 & 2	Identify predictors
Ausserhofer, 2013, USA [53]	NH	Frontline & Administration	Quantitative Survey	SOS	1	Psychometrics
Berlowitz, 2003, USA [54]	NH	Frontline & Administration	Quantitative Survey	Created Employee Survey	1	Culture Analysis
Bonner, 2009, USA [55]	NH	Frontline	Quantitative Survey	NHSOPSC	1 & 2	Culture Analysis
Bonner, 2008, N/A [51]	N/A	N/A	Literature Review	N/A	N/A	N/A
Brown, 2016, USA [70]	NH	Frontline	Quantitative Survey	NHSOPSC	1 & 2	Culture Analysis
Buljac-Samardzic, 2016, Netherlands [61]	NH & RH	Frontline	Quantitative Survey	SAQ	1	Culture Analysis
Castle, 2011, USA [62]	Hospital & NH	Frontline & Administration	Quantitative Survey	NHSOPSC	1 & 2	Culture Analysis
Castle, 2006, USA [63]	NH	Administration	Quantitative Survey	HSOPSC	1 & 2	Culture Analysis
Castle, 2006, USA [64]	NH	Administration	Quantitative Survey	HSOPSC	1 & 2	Culture Analysis
Castle, 2011, USA [65]	NH	Administration	Quantitative Survey	NHSOPSC	1 & 2	Culture Analysis
Castle, 2010, USA [66]	NH	Frontline & Administration	Quantitative Survey	NHSOPSC	1 & 2	Culture Analysis
Ginsburg, 2014, Canada [56]	Across settings, including NH	Frontline	Quantitative Survey	PSCS	1	Psychometrics
Handler, 2006, USA [67]	NH	Frontline	Quantitative Survey	PSCS	1 & 2	Culture Analysis
Hartmann, 2013, USA [68]	NH	Frontline	Quantitative Survey	CESARS	1 & 2	Psychometrics
Hughes, 2006, USA [69]	NH	Frontline	Quantitative Survey	HSOPSC	1 & 2	Culture Analysis
Mitchell, 2012, Canada [57]	Across settings, including LTC settings	Frontline & Administration	Quantitative Survey	Accreditation Canada’s Patient Safety Culture Tool	1 & 2	Culture Analysis
Scott-Cawiezell, 2006, USA [74]	NH	Frontline & Administration	MMR- Survey & Case Study	Selected HSOPSC survey items	1 & 2	Culture Analysis
Sheridan, 2014, Australia [59]	RH	Frontline	Qualitative Interviews	N/A	1 & 2	Culture Analysis
Thomas, 2012, USA [72]	NH	Administration	Quantitative Survey	NHSOPSC	1 & 2	Culture Analysis
Wagner, 2009, USA & Canada [58]	LTC settings including NH	Frontline	Quantitative Survey	HSOPSC	1 & 2	Culture Analysis
Wagner, 2013, Canada [59]	NH	Frontline	Quantitative Survey	CANE	2	Culture Analysis
Wagner, 2012, USA [73]	NH	Administration	Quantitative Survey	NHSOPSC	1 & 2	Culture Analysis
Wisniewski, 2007, USA [71]	NH	Frontline	Quantitative Survey	SAQ	1	Culture Analysis
Zuniga, 2013, Switzerland [60]	NH	Frontline	Quantitative Survey	NHSOPSC	1 & 2	Psychometrics

### Study characteristics

#### Available evidence

The scoping review first identified that this topic has not been extensively researched, with a relatively low number of texts exploring safety culture in care homes (*n* = 25). Nearly all texts were empirical research, with one literature review summarising the available empirical studies in 2008.

#### Context

Most of the empirical studies into care home safety culture were undertaken in the United States of America (USA) (*n* = 18) (e.g. [52–55]). There was much less empirical research into this topic from other countries, with a few articles from Canada (*n* = 3) [56–58], Australia (n = 1) [59], Switzerland (n = 1) [60] and the Netherlands (n = 1) [61]. This shows that there was significantly less European evidence exploring safety culture within care homes, and no studies available for the UK.

The vast majority of empirical studies were conducted in Nursing Homes settings alone (*n* = 18) (e.g. [62–69]). Some studies included multiple settings; in these instances Nursing Homes were included as one of the sites [56–58, 61, 62]. Two of the empirical studies undertook research in Residential Homes (*n* = 2). One of these studies focused on Residential Homes [59], whereas the other looked at Residential Homes against Nursing Homes [61].

#### Participants

Distinct participant categories were used to describe the focus of research across the literature. The first category was ‘Frontline staff’, which included all staff and healthcare professionals directly involved in patient care (e.g. Care Assistants, Nurses, Pharmacists, Doctors, Physiotherapists, Occupational Therapists, Activities Coordinators, Domestic staff). Within the review, most studies looked at the frontline staff alone (*n* = 12) (e.g. [70, 71]). The second category was ‘Administration staff’, which encompassed management and administration at all levels (e.g. Nursing Administrators, Care Home Managers, Directors, Owners). There were significantly fewer studies that looked at only the administration staff (*n* = 5) (e.g. [63–65, 72, 73]). However, many studies were found that looked at both frontline and administration staff in the same study (*n* = 7) (e.g. [54, 57, 74]). The final participant category was ‘Residents, service users or loved ones’, which was chosen to reflect the inclusion of these individuals’ contributions to and perspectives on safety culture. From this scoping review, there were no studies that considered the perspectives or experiences of residents, service users or their loved ones in relation to safety culture.

#### Research method

The scoping review also revealed a dominance of quantitative approaches to research methodology and methods. Quantitative surveys were utilized as the research method in the vast majority of the empirical studies (*n* = 22) (e.g. [55–58, 60–73]). In the quantitative papers, self-completion surveys by staff were used in all cases, and therefore findings reflected safety culture from the staff perspective.

Conversely, mixed methods approaches were much less commonly used, with the review finding just one study that used both qualitative case studies and a quantitative survey (*n* = 1) [74]. It was also clear that qualitative research methods have been underutilized within care home safety culture research, with only one qualitative interview study identified (n = 1) [59].

#### Theoretical orientation

The vast majority of papers sat within a functional structuralist paradigm (*n* = 23) (e.g. [52–55]). For most papers the functional structuralist theoretical position was implicit and not discussed in the paper, but these aligned with a positivist approach and appeared to understand safety culture as a real and tangible entity that could be measured and controlled [75]. Three papers used notions of Donabedian’s work on structure, process, outcome, and also clearly identified with functional structuralist underpinnings [55, 65, 72]. For example, Thomas et al. (2012) applied Donabedian’s model to the relationship of safety culture (structure), use of physical restraints (process) and residents who fell (outcome) [72]. Other theories used to explore safety culture included Berends’ safety culture model, which assumes the collective mental programming towards safety of a group of organisation members, [63] and the theory of high reliability organisations [68]. Two papers also explicitly discussed Schein’s levels of culture [56, 65].

One study was explicitly interpretive and explored safety culture through the use of qualitative interviews and aligned this with Reason’s Accident Causation model, which can be used to highlight how accidents happen in an organisation [59]. The final paper used a mixed methods approach and appeared to be underpinned by pragmatic assumptions [74].

#### Measurement tool

As quantitative surveys were the dominant method adopted across the identified articles, the scoping review also examined the measurement tools used to assess safety culture. The HSOPSC, or the care home adaptation of this (Nursing Home Survey on Patient Safety Culture [NHSOPSC]), were most frequently used to assess patient safety culture (*n* = 14) (e.g. [62–66]). This is also the dominant culture survey used within hospital settings, where it was originally developed and validated. However, the literature search found only one study that was attempting to test the validity and reliability of this measurement tool for use in Nursing Home settings [60].

Various other surveys were used, but again there was proportionately little evidence aiming to validate these measurement tools and test psychometrics for use in care home settings.

Alternative survey tools used included the Safety Attitudes Questionnaire (SAQ) (*n* = 2) [61, 71], Communicating About Nursing Errors survey (CANE) (*n* = 1), Accreditation Canada’s Patient Safety Culture Tool (n = 1) [57], Community Living Centres Employee Survey on Attitudes about Resident Safety (CESARS) (n = 1) [68], Patient Safety Climate Survey (PSCS) (n = 2) [56, 67], Safety Organizing Scale (SOS) (*n* = 1) [53] and a specifically developed employee survey (n = 1) [54].

#### Level of analysis

The level of analysis of each empirical study was identified. Six empirical studies looked at the most superficial level of ‘Artifacts’, considering only the visible surface manifestations of safety culture (*n* = 5) (e.g. [53, 54, 61]). One study used the second level of analysis (beliefs and values) to explore safety culture (n = 1). Overall, the majority of studies looked at both level 1 and 2 together, considering the superficial artifacts alongside beliefs and values that could be captured within both survey and interview data (*n* = 18) (e.g. [67–69]). The third level “Basic Underlying Assumptions” represents the deepest level for analyzing culture, and is only researched through the use of qualitative methods. Although the review found some qualitative studies, no studies that looked at this deepest level of safety culture were identified.

#### Focus

The scoping review also aimed to look at the focus of the research and what it aimed to achieve. The majority of empirical research was involved in the direct analysis of safety culture (*n* = 19) (e.g. [61–67]), often carried out using predesigned safety culture surveys that had been developed and validated within hospital care settings. Four studies looked at psychometrics and attempted to assess the validity and reliability of such measures for use in care home settings (*n* = 4) [53, 56, 60, 68]. The one remaining empirical study focused on the identification of predicting factors for safety culture (n = 1) [52].

## Discussion

### The research context

Over the past decade there has been a clear increase in the amount of studies exploring safety culture in care homes, with all but two of the identified articles published after 2006.

In the early 2000s safety culture became a priority area in the USA following the landmark reports “to Err is Human” and “Crossing the Quality Chasm” [2, 11]. Soon after this the HSOPSC was developed and published in 2004, and was largely applied to acute inpatient settings [63]. Two early studies were undertaken by Castle (2006) and Castle et al. (2006) applying the HSOPSC to nursing home settings [63, 64]. However, safety culture in care homes has still not been widely investigated, and therefore the available evidence is limited.

The identified studies were found to not be representative of care home settings globally. This appeared to be due to the dominance of studies from the USA (*n* = 18), which therefore reflected the prevalence of Nursing Homes across the USA care home sector. Although this evidence has expanded the evidence base, the implication of the dominance of USA papers is that the available research into safety culture does not adequately reflect variations in care homes across countries. For example, within Europe alone there has been vast variation in typology noted, with four categories that reflect differences in the use, service provision resources, financing and privatization of care homes, all of which may influence safety culture in these settings [76]. Moreover, the international prevalence measurement of care problems index, which provides an audit of the prevalence of care problems across the Netherlands, Austria, New Zealand and Switzerland, has found varying prevalence of care problems across countries [77, 78]. It can therefore be assumed that across countries there will be differences not only in the service typology, but also variation in care problems that these settings face.

The vast majority of studies on this topic included Nursing Homes as a research setting (*n* = 24), and there was proportionately much less research into safety culture within Residential Homes (n = 2). The review provides an insight across the available evidence and highlights that the context of Nursing Homes has been predominantly used to investigate this topic. Alternative Residential Home settings have therefore been under investigated. This is particularly noteworthy for countries like the UK, whose care home sector is dominated by Residential Homes [31–33], as these settings are distinct and face different challenges in terms of safety and safety culture. The main difference is that Residential Homes rely on an unregulated workforce to manage and deliver care [42]. This unregistered and unregulated workforce has been found to complete many tasks that were previously undertaken by registered staff [79–82]. However, it is acknowledged that they are often under qualified for these roles, with almost 40% of unregistered staff undertaking direct care roles having no qualifications [42]. Therefore, Residential Homes face significant challenges in meeting the needs of these vulnerable older people with highly complex physical, emotional, and social, needs [32–34]. As such, there should be studies within Residential Homes that can investigate the impact of these factors on safety culture.

In terms of context, the scoping review has presented a specific knowledge gap in terms of safety culture across countries and the available evidence within Residential Homes. Overall, more robust evidence into care home safety culture across countries, care home typology and settings and particularly residential homes should be made available.

### Dominant theoretical underpinnings and methodology

The empirical study of safety culture in care homes had a dominant functional/structuralist approach, with quantitative approaches used within the vast majority of the identified studies. In particular, self-completion cross-sectional surveys were used to capture safety culture in care homes. However, this methodological approach to assessing organisational culture has been criticised due to the subjective nature of responses, which may not reflect the complexity of influences on culture [75]. Although survey tools are predominantly used to capture safety and organisational culture, a further disadvantage of these functional/structuralist assumptions is that these methodological approaches do not lend themselves to exploration of deeper meaning and can therefore only capture these more superficial levels [18–20, 75]. This is a particular limitation of the evidence, as Parker (2000) and Schein (1988, 2004, 2010) both warn that the more superficial levels of analysis can be misleading, and do not always allow for the deeper organisational culture to be explored, particularly when data is collected via surveys [18–20, 75].

The scoping review found that across the studies, interpretive approaches were rarely used, and that no ethnographic studies had been undertaken. Although investigation at more superficial levels can provide insight into safety culture within care homes, Schein (1988, 2004, 2010) explains that we can only truly understand organisational culture if this deeper level of culture is explored [18–20]. As such, the scoping review revealed a knowledge gap in the exploration of safety culture in care homes, with no studies achieving this level of analysis. Ethnographic methods have been highlighted as an approach that can explore the complexity of organisational culture at all levels, and therefore presents a potential method for addressing this gap in the evidence [75].

The scoping review has highlighted a dominance of quantitative survey measures, with little use of interpretive approaches and no ethnographic investigation of safety culture in care homes. This influence of these approaches is that only superficial levels of safety culture can be analysed, with all studies looking at level 1 or 2. Schein (1988, 2004, 2010) argues that these superficial studies are unable to deeply explore the organisational culture, and to fully understand organisational culture it is necessary to understand the deep “Basic Underpinning Assumptions” at level 3 of analysis [18–20]. As there were no studies available that captured this third level of analysis, it could be argued that the available literature has been unable to deeply explore the complexity of safety culture within care home settings.

### Appropriateness and validation of tools

A variety of different measurement tools were used to explore this topic, with the HSOPSC and NHSOPSC used in the majority of studies. The scoping review found that although this measurement tool has been used within care home settings, only one study was available that looked at the validity and reliability of this for use within Nursing Homes [60]. This is important as most safety culture tools originated from hospital contexts and have been predominantly tested and validated within mainstream care settings. Therefore, without robust validation of these measurement tools we cannot be sure that they have the same level of reliability or validity within care home settings, which are vastly different to acute hospitals.

### Inclusive participant groups

Finally, the scoping review found that all of the research into safety culture in care homes focused on staff in this setting. This is not unique to the care home literature, as staff are predominantly the focus of research into organisational and safety culture [13, 14]. However, care homes do present a distinctly different care setting, as they are also a person’s home where they receive 24-h care, often in the last years of life [32]. As such, it could be argued that residents in this setting are also part of the organisation and consequently contribute to the organisational and safety culture. These recursive relationships have been found to exist within healthcare, and it is therefore argued that patients and service users should be included within research into organisational culture [14]. The scoping review found that no studies into safety culture in care homes used residents, service users or their loved ones as participants. This consequently highlights that the inclusion of these groups is a final gap within the literature. As care homes are not only a place of care, but also the individual’s home, this may be a potential oversight of the research into safety culture in this context. This could be particularly important for non-mainstream social care settings, such as long-term care, where patients, service-users, residents and their families make up a central part of the organisation.

### Key gaps

This scoping review has revealed four key gaps in the evidence base around safety culture in care homes. The first is that overall this topic is not extensively explored and that more research into safety culture in care homes, and the validation of culture surveys in care homes, is required. Secondly, more research that aligns with an interpretive philosophy is required. This will allow for the deeper levels of culture to be explored. Future studies into culture in care homes should therefore use qualitative methods, such as ethnography. Thirdly, no research into safety culture in care homes included the perspectives of service users. Due to the nature of care homes as a long term care setting, residents and their loved ones should be included in the study of safety culture in this setting, as these groups make up an essential part of the organisation. Finally, the review revealed proportionately fewer research studies in residential care home settings; more research is therefore required into safety culture in these contexts.

### Strengths and limitations

This review had a number of key strengths, including its broad scope in attempting to identify the extent, range and nature of available research into safety culture in care homes [43]. Additionally, the use of scoping review methodology allowed key gaps to be identified, as well as limitations of the available evidence [43, 44]. Overall, the review was able to provide an overview of the available research, and highlight key aspects as the focus of future research in the exploration of safety culture within this setting. Furthermore, the literature search followed a systematic and robust process, with two reviewers used for a large proportion of the full text analysis.

A limitation of the review is that alternative media, such as reports and books, were not included in the literature searching process. Moreover, a narrow search string was used, which is a further limitation of the scoping review methodology as it is possible that some relevant papers were missed [46]. As most of the available research was based in the USA, the study findings into safety culture will reflect the USA context of healthcare services. As such, this research should not be generalised to countries that have different approaches to adult social care in terms of service provision and funding. A final limitation of this review was that it did not attempt to assess quality of the evidence included or aggregate findings and instead provided a broad overview of the subject area [43, 44].

## Conclusion

Overall, safety culture in care homes is a topic that has not been extensively researched, and this review has identified a number of gaps in the current evidence base. The collective evidence demonstrated that safety culture research largely takes place in Nursing Homes rather than Residential Homes, where a significant proportion of the older population in the UK currently reside [31]. Moreover, the majority of available evidence is based in the USA and therefore does not capture UK specific issues around access, funding and service provision. The empirical evidence predominantly used quantitative measures, and therefore the deeper levels of culture have not been captured in the evidence base, despite this being identified as an essential factor in providing a comprehensive understanding of the complexity of organisational cultures [18–20]. The key points that have been highlighted from this review have therefore demonstrated where future research in this topic area requires focus and development.

## Abbreviations

CANE: Communicating About Nursing Errors survey
CESARS: Community Living Centres Employee Survey on Attitudes about Resident Safety
HSOPSC: Hospital Survey on Patient Safety Culture
NH: Nursing Home
NHSOPSC: Nursing Home Survey on Patient Safety Culture
PSCS: Patient Safety Climate Survey
RH: Residential Home
SAQ: Safety Attitudes Questionnaire
SOS: Safety Organizing Scale
USA: United States of America
